# Comparative efficacy of biologics as monotherapy and in combination with methotrexate on patient reported outcomes (PROs) in rheumatoid arthritis patients with an inadequate response to conventional DMARDs – a systematic review and network meta-analysis

**DOI:** 10.1186/1477-7525-12-102

**Published:** 2014-07-03

**Authors:** Jeroen P Jansen, Felicity Buckley, Fred Dejonckheere, Sarika Ogale

**Affiliations:** 1 Previously of Mapi Group, Boston, MA, USA; 2 Tufts University School of Medicine, Boston, MA, USA; 3 Mapi Group, 180 Canal Street, Suite 503, Boston, MA 02114, USA; 4 F. Hoffman-La Roche, Basel, Switzerland; 5 Genentech, South San Francisco, CA, USA

**Keywords:** Rheumatoid arthritis, Biologics, Patient reported outcomes, Network meta-analysis, Indirect comparison

## Abstract

**Objective:**

To compare biologics as monotherapy or in combination with methotrexate (MTX) in terms of patient reported outcomes (PROs) in RA patients with an inadequate response to conventional DMARDs (DMARD-IR).

**Methods:**

With a systematic literature review 17 RCTs were identified that evaluated adalimumab, certolizumab pegol, etanercept, golimumab, infliximab, abatacept, anakinra or tocilizumab. Treatment effects in terms of pain (0-100 mm), patient’s global assessment of disease activity (PGA; 0-100 mm), Health Assessment-Questionnaire (HAQ) disability index (DI; 0–3), and the physical component summary (PCS) of the SF36 Health Survey (0–100) at 24 weeks were combined by means of Bayesian network meta-analyses.

**Results:**

With tocilizumab monotherapy, greater improvements in pain (difference = -11.1; (95% Credible Interval -21.3, -0.1)) and PGA (-10.3 (-20.4, 0.8)) were observed than with aTNF monotherapy. Tocilizumab was at least as efficacious as aTNF in HAQ-DI improvements (-0.16; (-0.37, 0.05)). aTNF + MTX (-17.9 (-23.1, -13.0) & -19.1 (-24.2, -14.4)), abatacept + MTX (-23.0 (-47.3, 1. 5) & -13.6 (-28.4, 2.0)) and tocilizumab + MTX (-16.0 (-26.3, -6.3) & -15.1 (-25.1, -5.7)) showed comparable reductions in pain and PGA relative to MTX. Efficacy of anakinra + MTX was much smaller as compared to other biologics. The greatest improvements in HAQ-DI relative to MTX were observed with aTNF + MTX (-0.30 (-0.37, -0.22)) and tocilizumab + MTX (-0.27 (-0.42, -0.12)), followed by abatacept + MTX (-0.21 (-0.37, -0.05)) and anakinra + MTX (-0.11 (-0.26, 0.05)). The improvements in SF36-PCS with abatacept + MTX, aTNF + MTX and tocilizumab + MTX were comparable. There is a >90% probability that aTNF + MTX results in a greater improvement in pain (-12.4), PGA (-16.1) and HAQ-DI (-0.21) than aTNF as monotherapy. Efficacy of tocilizumab + MTX showed comparable improvements in PROs as tocilizumab monotherapy.

**Conclusions:**

Based on a network meta-analysis involving indirect comparison of trial findings, the following observations were made for DMARD-IR patients. In monotherapy, tocilizumab was associated with a greater improvement in pain and self-reported disease activity than aTNF, and was at least as efficacious regarding functional ability. The improvements in PROs with aTNF, abatacept and tocilizumab in combination with MTX were comparable. Improvements in PROs with tocilizumab as monotherapy were similar to that of tocilizumab + MTX, whereas aTNF as monotherapy was likely to be less efficacious than aTNF + MTX.

## Background

Rheumatoid arthritis (RA) is a chronic inflammatory joint disorder characterised by joint stiffness, swelling, and pain, and can have a profound impact on a patient’s health related quality of life [1,2]. As such, the goals of treatment of RA are not only symptom relief, reduction in disease activity, and reduction in the rate of joint damage, but also improvement in physical functioning and well-being from the patient’s perspective [3,4].

The European League Against Rheumatism (EULAR), American College of Rheumatology (ACR), and Outcomes Measures in Rheumatology (OMERACT) have outlined the importance of patient reported outcomes (PROs) in addition to physician assessed outcomes for the complete assessment of progression of disease and the evaluation of the effectiveness of RA treatment [5]. PROs used for the assessment of treatments in RA clinical trials typically include pain, patient's global assessment of disease activity (PGA), and the general health measures Health Assessment-Questionnaire (HAQ) disability index (DI) and Medical Outcomes Study Short Form 36 Health Survey (SF36) [6-8].

Patients who are intolerant or show an inadequate response (IR) to traditional disease-modifying anti-rheumatic drugs (DMARDs) are often treated with a biologic agent. For DMARD-IR patients, biologics are usually combined with traditional DMARDs, primarily methotrexate (MTX), but some biologics are approved and have been shown to be efficacious as monotherapy as well [9-11]. In real life, approximately one-third of RA patients on biologics are on monotherapy [12-14].

Given the number of the alternative biologic treatment options for the DMARD-IR RA population, clinicians are faced with a challenging choice regarding the optimal treatment. There is no randomized controlled trial (RCT) that evaluates all approved biologics simultaneously to help answer this question. The available evidence base consists of multiple placebo controlled trials and some active head-to-head comparisons. Network meta-analysis has been introduced, as a generalisation of pair-wise meta-analysis, to simultaneously synthesize the different RCTs evaluating different biologics and perform indirect comparisons in the absence of head-to-head studies. In the past few years several network meta-analysis of biologic treatments for RA have been published [15-22]. However, currently there is no network meta-analysis that compares the treatment effects of combination therapy and monotherapy regarding PROs.

The objective of the current study was to compare the efficacy of biologic DMARDs used as monotherapy or in combination with MTX in terms of pain, self-reported disease activity, functional ability, and overall health related Quality of Life (HRQoL) among DMARD-IR RA patients based on currently available evidence from RCTs.

## Methods

### Identification and selection of studies and data extraction

The following criteria for considering published studies for review were used:

• *Population of interest*: DMARD-IR RA patients.

• *Interventions*: tocilizumab, TNF-blockers, abatacept, and anakinra in their usual dose, alone and in combination with conventional DMARDs. Rituximab was not considered because its label is restricted to TNF-IR patients. Tofacitinib was not included because it was not approved at the time of this study.

• *Comparisons*: Placebo or one of the regimes described under interventions. Comparisons of different dosages of the same intervention only, or comparison of the same interventions with different background treatments were excluded.

• *Outcomes/endpoints:* HAQ-DI, Pain, PGA, SF36, and fatigue.

• *Study design*: randomized controlled trials

• *Exclusion*: Studies with solely Asian patients, and non-English language publications were excluded.

The pre-defined search strategy of the Medline, Embase, and Cochrane databases used terms related to RA, biologics, and RCTs to allow for a systematic search of studies published between 1990 and April 2012 (See Appendix for search strategy). Titles and abstracts were screened to ascertain whether studies met predefined selection criteria. Studies that either met the criteria or for which it was unclear were further screened using the full text report.

For each identified study that met the selection criteria, details were extracted on study design, study population characteristics, study quality according to the Jadad criteria [23], interventions, and the outcomes pain, PGA, HAQ-DI, and SF36. Pain and PGA were assessed on 0 to 100 mm visual analog scale (VAS); higher scores reflect greater pain and disease activity and minimum clinically important differences (MCIDs) are ≥10 mm increase from baseline [24-28]. HAQ-DI assesses the level of an individual’s functional ability and scores range from 0 to 3; higher scores indicate more severe disability and the MCID is a ≥ 0.22 points increase [25]. The SF36 yields 8 domain scores which are summarized in a physical health component summary (PCS) score and mental health component summary (MCS) score. The scale ranges from 0 to 100 with higher scores reflecting greater HRQoL. Improvements of ≥ 5 points from baseline represent a MCID [7,8].

### Network meta-analysis

To synthesize the results of the included studies, Bayesian network meta-analysis models were used [29-32]. For the analysis we grouped the different aTNFs because previous analysis demonstrated that the different aTNFs are exchangeable [19,20]. Within a Bayesian framework, analysis involves data, a likelihood distribution, a model with parameters, and prior distributions for these parameters [33]. A regression model with a normal likelihood distribution relates the data from the individual studies to basic parameters reflecting the (pooled) treatment effect of each intervention compared to placebo. Based on these basic parameters, the relative efficacy between each of the compared biologics, as monotherapy and combination was calculated.

Both fixed and random effects models were considered and were compared regarding the goodness-of-fit to the data, calculated as the posterior mean residual deviance. The deviance information criterion (DIC) provides a measure of model fit that penalizes model complexity [34]. The random effects model resulted in the lowest DIC, and was considered appropriate for the synthesis of the available evidence.

To avoid influence of the prior distributions required for the Bayesian analyses on results, non-informative prior distributions were used. Prior distributions of the treatment effects relative to placebo were normal distributions with mean 0 and a variance of 10,000. A uniform distribution with range of 0–20 (pain, PGA, SF36) and 0–6 (HAQ) was used for the prior distribution of heterogeneity needed for the random effects analyses. WinBUGS statistical software was used for the analyses [35]. The results of the network meta-analysis provide us with posterior distributions of treatment effects of each treatment versus placebo in terms of difference in change from baseline. In order to transform these difference measures into an expected change from baseline with each treatment, the effect estimates of each regimen relative to placebo were combined with the average change from baseline with placebo across studies. The posterior distributions of the treatment effect (i.e. difference in change from baseline) and expected change from baseline by treatment were summarized with the median and 95% credible intervals (95% CI) reflecting the range of true underlying effects with 95% probability. Based on the posterior distributions of relative treatment effects the probability that a certain intervention was more efficacious than a competitor was calculated, as well the probability that each treatment ranks 1^st^, 2^nd^, 3^rd^, etc. The latter findings were expressed with rankograms.

## Results

### Study identification

**Figure 1:**
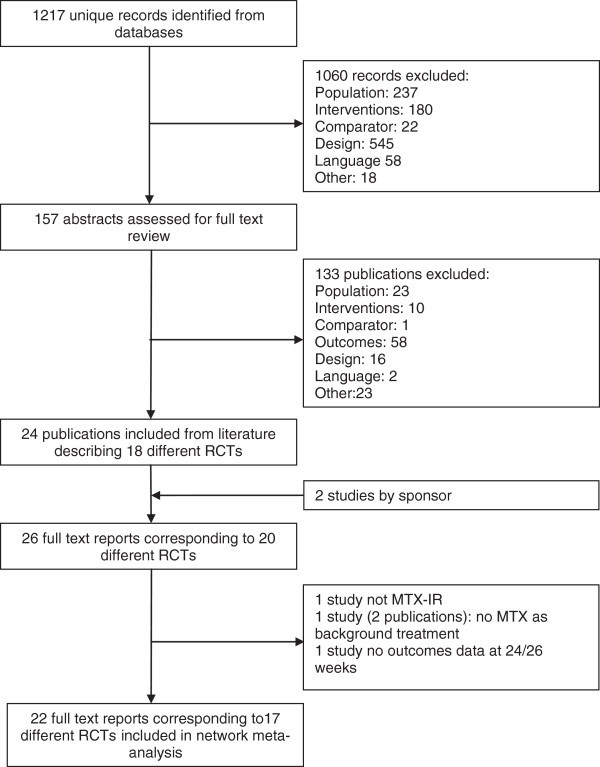
Flow diagram of study identification and selection.

The literature search resulted in 1,217 unique, potentially relevant citations, of which abstract review excluded 1,060 (87%) (Figure 1). Of the remaining 157 retrieved full text publications, 133 (11%) were excluded through the full-text review. A total of 26 full text reports corresponding to 20 different RCTs, including 2 studies provided by Roche (ACT-RAY and ADACTA) met the selection criteria [9-11,36-56]. These 2 latest studies were not published at the time of the data cut, but were considered crucial for the evidence network.

### Evidence base

Most of the trials were multi-centred and included patients predominantly from Europe and North America. The RCTs were generally considered to be good quality (Jadad score range 3–5). All included trials were double blind with appropriate description of drop out of subjects, although the method of randomisation and blinding was not always reported. The majority of the studies included adult patients with diagnosis of RA based on the ACR 1987 revised classification criteria. All studies included DMARD-IR patients. Although the definition of DMARD-IR varied somewhat between the studies, it was most commonly defined as patients with active disease despite of previous treatment with traditional DMARDs. The traditional DMARD was often specified to be MTX, although in fewer studies it was unspecified. Other definitions included inadequate response to prior DMARDs, or patients who are either intolerant to MTX, or the use of MTX is inappropriate. The TEMPO trial included patients who were non-responders to DMARDs but disqualified patients who had failed MTX treatment [52]. Given this difference, the study was excluded from the network meta-analysis. The definitions of active disease varied in terms of the minimum levels of ESR (10 mm/h, 28 mm/h) and CRP (2 mg/dl, 1 mg/dl, 1.5 mg/dl, 7 mg/ml), as well as in terms of the minimum number of required tender [6-12] and swollen [6-12] joints. Not all studies reported whether RA disease duration and DMARD treatment duration determined eligibility.

In RCTs evaluating the efficacy of biologics in combination with a traditional DMARD, MTX was the background treatment of choice, except for the study by Combe et al. in which sulfasalazine was used [37,38]. To allow a valid indirect comparison between treatments with the network meta-analysis, this study was excluded as well. The study by Schiff et al. was also excluded because no results at 24 weeks were provided for the outcomes of interest [48].

**Figure 2:**
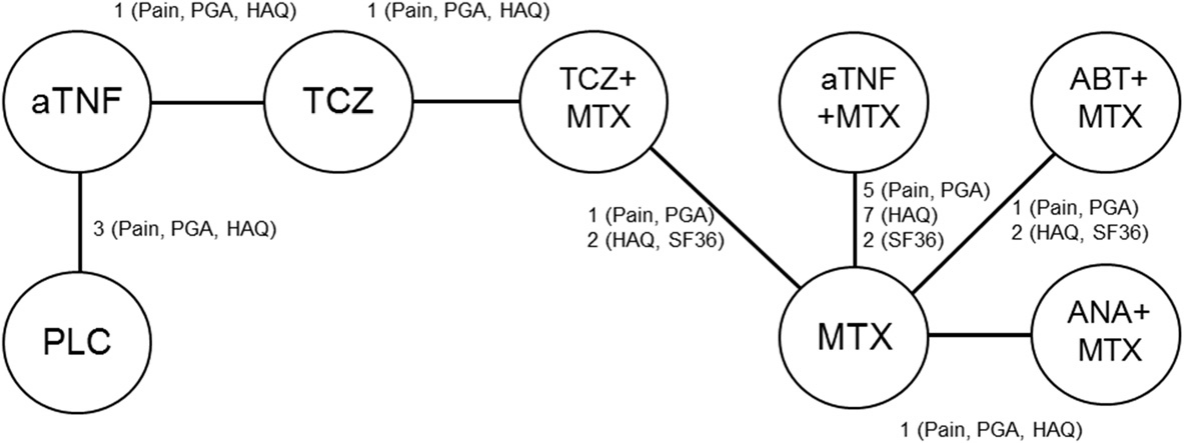
Network of randomized controlled trials evaluating agents for DMARD-IR RA patients in terms of PROs at 24 weeks.

Thirteen studies, including ACT-RAY and ADACTA, provided outcome data for pain and PGA [9,11,36,39,41,44,49-51,54,55]. All seventeen studies provided information on HAQ-DI. Eight studies (including ADACTA) provided information on the SF36 PFS [9,40,44,47,49-51], but 2 of these studies (ADACTA and Matthias 2000) could not be used for the network meta-analysis because these studies could not be linked to the network of RCTs. The number of studies providing information on the SF36 MCS was too limited to allow network meta-analysis. Nine studies (including ADACTA) reported fatigue as an outcome measure, but given differences in the instruments used (i.e. Functional Assessment of Chronic Illness Therapy-Fatigue (FACIT-F), Fatigue Assessment Scale (FAS), and Fatigue VAS) a network meta-analysis was not considered feasible [39,40,43,47,49-51,56].In Figure 2 the network of the 17 RCTs is presented where each line between nodes reflects the available direct comparisons. By means of network meta-analysis a treatment effect of each intervention relative to another that is part of the same network can be obtained.

Table 1 provides information on the study and patient characteristics of the 17 RCTs used for the network meta-analysis. The mean age in the study arms ranged from 48 to 57. Female patients were predominant; the proportion of women in the study arms ranged from 66% to 90%. Disease duration ranged from 4.5 to 13 years, swollen joint count ranged from 11.3 to 21.9, and tender joint count ranged from 13 to 35.5. The reported ESR ranged from 25 to 56.1 mm/1 hr, CRP varied between 8 and 52.6, and rheumatoid factor positivity ranged from 77% to 100%. Despite some variation in patient characteristics across studies (i.e. duration of disease, lower swollen and tender joint count, and lower CRP), there were no observed systematic differences across the different types of direct comparisons, indicating the feasibility of the network meta-analysis.

**Table 1:** Study and patient baseline characteristics of studies included in the network meta-analysis

**Study**	**Interventions**	**Number of patients**	**AGE (years)**	**Female (%)**	**Disease duration (years)**	**SJC (0–66)**	**TJC (0–68)**	**ESR (mm/hr)**	**CRP (mg/L)**	**RF + ve number (%)**
Kremer [44]	ABT 10 mg/kg Q4W + MTX	115	56	75	10	21.3	30.8	NR	29	90
	Placebo + MTX	119	55	66	9	21.8	29.2	NR	32	90
Kremer [43], Russell [47]	ABT 10 mg/kg Q4W + MTX	433	52	78	9	21.4	31	NR	33	82
	Placebo + MTX	219	50	82	9	22.1	32.3	NR	28	79
Cohen [36]	ANA 100 mg QD + MTX	250	56	79	11	20.1	26.8	41.5	27	76
	Placebo + MTX	251	57	75	10	20	24.5	42.9	26	78
Maini [46], Lipsky [45] (ATTRACT)	IFX 3 mg/kg Q8W + MTX	86	54	81	10	22	32	49	39	84
	Placebo + MTX	88	51	80	11	21	31	49	40	77
Keystone [41], Yount [56]	ADA 40 mg QOW + MTX	207	56	76	11	19.3	27.3	NR	18	82
	Placebo + MTX	200	56	73	11	19	28.1	NR	18	90
Weinblatt [54], Yount [56] (ARMADA)	ADA 40 mg QOW + MTX	67	57	75	12	17.3	28	NR	21	NR
	Placebo + MTX	62	56	82	11	16.9	28.7	NR	31	NR
Van de Putte [11]	ADA 40 mg QOW	113	53	80	11	20.5	33.7	55.8	52.6	80
	Placebo	110	54	77	12	19.8	35.5	56.1	57	82
Strand [51], (RAPID 1)	CTZ 200 mg QOW + MTX	393	51	82	6	9.9 ^M^	12.4 ^M^	43.5 ^M^	16 ^M^	80
	Placebo + MTX	199	52	84	6	9.7 ^M^	13 ^M^	45 ^M^	16 ^M^	83
Smolen [50], Strand [52], (RAPID 2)	CTZ 200 mg QOW + MTX	246	52	84	6	20.5	30.1	43.7	14.2	78
	Placebo + MTX	127	52	84	6	21.9	30.4	40.8	13.5	78
Fleischmann [39] (FAST4WARD)	CTZ 400 mg Q4W	111	53	78	9	21.2	29.6	30.9	11.6	100
	Placebo	109	55	89	10	19.9	28.3	35.6	11.3	100
Weinblatt [55]	ETN 25 mg BW + MTX	59	48	90	13	20	28	25	22	84
	Placebo + MTX	30	53	73	13	17	28	36	26	90
Moreland [10], Mathias [9]	ETN 25 mg BW	78	53	74	11	25	33	35	47	79
	Placebo	80	51	76	12	25	35	39	41	79
Keystone [42] (GO-FORWARD)	GLB 50 mg Q4W + MTX	89	52 ^M^	81	4.5 ^M^	13 ^M^	26 ^M^	NR	10 ^M^	81
	Placebo + MTX	133	52 ^M^	82	6.5 ^M^	12 ^M^	21 ^M^	NR	8 ^M^	81
Genovese [40] (TOWARD)	TCZ 8 mg/kg Q4W + MTX	803	53	81	10	19.7	30.1	48.2	26	NR
	Placebo + MTX	413	54	84	10	18.7	29.1	49.2	26	NR
Smolen [49] (OPTION)	TCZ 8 mg/kg Q4W + MTX	205	51	NR	8	19.5	31.9	51.2	26	83
	Placebo + MTX	204	51	NR	8	20.7	32.8	49.7	24	71
ACT-RAY	TCZ 8 mg/kg Q4W + MTX	277	53	81.9	8.2	14.4	25.8	39.9	NR	NR
	TCZ 8 mg/kg Q4W	276	53.6	78.6	8.3	15.3	26.6	39.6	NR	NR
ADACTA	TCZ 8 mg/kg	163	54.4	79	7.3	11.3	15.9	50.5	26	NR
	ADA 40 mg	162	53.3	82	6.3	12.4	16.5	45.5	25	NR

M = median; NR = not reported; SJC = swollen joint count; TJC = tender joint count; ESR = erythrocyte sedimentation rate; CRP = C-reactive protein; RF + ve = Rheumatoid factor positive ABT = abatacept; ANA = anakinra; IFX = infliximab; ADA = adalimumab; CTZ = certolizumab pegol; ETN = etanercept; GLB = golimumab; TCZ = tocilizumab; MTX = methotrexate.

### Monotherapy

In Tables 2, 3, 4 and 5 the results of the network meta-analysis are presented. Each cell presents the difference in change from baseline for the outcome of interest 24 weeks with the intervention (in the rows) relative to a comparator (in the column). Individual study results are provided in Additional file 1: Table S1.

**Table 2:** Treatment effects for all contrast in terms of pain (pain VAS) along with 95% credible interval and probability that treatment is better than the comparator

**Intervention**		**Comparator**							
		**Placebo**	**MTX**	**aTNF**	**Tocilizumab**	**aTNF + MTX**	**Abatacept + MTX**	**Anakinra + MTX**	**Tocilizumab + MTX**
Placebo	Estimate	0	14.71	20.17	31.28	32.53	37.63	22.00	30.71
	95% CrI		(-3.85, 33.43)	(12.33, 29.73)	(18.69, 45.21)	(13.46, 52.09)	(6.71, 67.22)	(0.86, 42.52)	(15.14, 46.97)
	P(better)		5%	<1%	<1%	<1%	1%	2%	<1%
MTX	Estimate	-14.71	0	5.42	16.55	17.85	22.98	7.29	15.97
	95% CrI	(-33.43, 3.85)		(-10.37, 24.07)	(3.81, 31.31)	(13.02, 23.08)	(-1.54, 47.31)	(-2.54, 16.69)	(6.26, 26.34)
	P(better)	95%		22%	1%	<1%	3%	5%	<1%
aTNF	Estimate	-20.17	-5.42	0	11.09	12.40	17.27	1.84	10.60
	95% CrI	(-29.73, -12.33)	(-24.07, 10.37)		(0.09, 21.3)	(-6.63, 29.01)	(-13.17, 45.84)	(-19.57, 19.71)	(-4.53, 23.59)
	P(better)	>99%	78%		2%	7%	12%	40%	6%
Tocilizumab	Estimate	-31.28	-16.55	-11.09	0	1.30	6.23	-9.29	-0.56
	95% CrI	(-45.21, -18.69)	(-31.31, -3.81)	(-21.3, -0.09)		(-13.98, 15.15)	(-21.98, 33.48)	(-27.22, 6.19)	(-10.64, 8.41)
	P(better)	>99%	99%	98%		41%	33%	91%	56%
aTNF + MTX	Estimate	-32.53	-17.85	-12.40	-1.30	0	5.06	-10.60	-1.85
	95% CrI	(-52.09, -13.46)	(-23.08, -13.02)	(-29.01, 6.63)	(-15.15, 13.98)		(-19.92, 29.83)	(-21.84, -0.05)	(-12.93, 9.48)
	P(better)	>99%	>99%	93%	59%		35%	98%	65%
Abatacept + MTX	Estimate	-37.63	-22.98	-17.27	-6.23	-5.06	0	-15.61	-6.93
	95% CrI	(-67.22, -6.71)	(-47.31, 1.54)	(-45.84, 13.17)	(-33.48, 21.98)	(-29.83, 19.92)		(-42.08, 10.48)	(-33.04, 19.51)
	P(better)	99%	97%	88%	67%	65%		89%	70%
Anakinra + MTX	Estimate	-22.00	-7.29	-1.84	9.29	10.60	15.61	0	8.73
	95% CrI	(-42.52, -0.86)	(-16.69, 2.54)	(-19.71, 19.57)	(-6.19, 27.22)	(0.05, 21.84)	(-10.48, 42.08)		(-4.56, 23.05)
	P(better)	98%	95%	60%	9%	2%	11%		7%
Tocilizumab + MTX	Estimate	-30.71	-15.97	-10.60	0.56	1.85	6.93	-8.73	0
	95% CrI	(-46.97, -15.14)	(-26.34, -6.26)	(-23.59, 4.53)	(-8.41, 10.64)	(-9.48, 12.93)	(-19.51, 33.04)	(-23.05, 4.56)	
	P(better)	>99%	>99%	94%	44%	35%	30%	93%	

P(better) = Probability that treatment (in row) is showing greater efficacy than comparator (in column); CrI = credible interval; aTNF = Anti-tumor necrosis factor.

**Table 3:** Treatment effects for all contrast in terms of patient global assessment (PGA VAS) along with 95% credible interval and probability that treatment is better than the comparator

**Intervention**		**Comparator**							
		**Placebo**	**MTX**	**aTNF**	**Tocilizumab**	**aTNF + MTX**	**Abatacept + MTX**	**Anakinra + MTX**	**Tocilizumab + MTX**
Placebo	Estimate	0	14.32	17.35	27.69	33.49	27.98	23.04	29.43
	95% CrI		(-4.66, 32)	(10.63, 25.39)	(15.06, 40.53)	(14.01, 51.89)	(3.21, 50.46)	(2.06, 42.8)	(13.49, 44.66)
	P(better)		5%	<1%	<1%	<1%	2%	2%	<1%
MTX	Estimate	-14.32	0	2.91	13.22	19.05	13.62	8.72	15.06
	95% CrI	(-32, 4.66)		(-12.59, 21.31)	(0.76, 27.44)	(14.36, 24.21)	(-1.97, 28.4)	(-0.37, 17.84)	(5.66, 25.14)
	P(better)	95%		34%	2%	<1%	4%	3%	1%
aTNF	Estimate	-17.35	-2.91	0	10.29	16.09	10.60	5.83	12.00
	95% CrI	(-25.39, -10.63)	(-21.31, 12.59)		(-0.8, 20.37)	(-2.65, 32.53)	(-13.52, 31.58)	(-15.01, 23.52)	(-2.94, 24.98)
	P(better)	>99%	66%		3%	4%	16%	23%	4%
Tocilizumab	Estimate	-27.69	-13.22	-10.29	0	5.76	0.27	-4.46	1.71
	95% CrI	(-40.53, -15.06)	(-27.44, -0.76)	(-20.37, 0.80)		(-8.91, 19.33)	(-20.68, 19.62)	(-21.41, 10.72)	(-7.84, 10.29)
	P(better)	>99%	98%	97%		17%	49%	77%	30%
aTNF + MTX	Estimate	-33.49	-19.05	-16.09	-5.76	0	-5.40	-10.34	-4.00
	95% CrI	(-51.89, -14.01)	(-24.21, -14.36)	(-32.53, 2.65)	(-19.33, 8.91)		(-22, 10.07)	(-20.94, -0.25)	(-14.82, 6.97)
	P(better)	>99%	>99%	96%	83%		76%	98%	81%
Abatacept + MTX	Estimate	-27.98	-13.62	-10.60	-0.27	5.40	0	-4.86	1.39
	95% CrI	(-50.46, -3.21)	(-28.4, 1.97)	(-31.58, 13.52)	(-19.62, 20.68)	(-10.07, 22)		(-22.16, 13.21)	(-16.06, 20.05)
	P(better)	98%	96%	84%	51%	24%		71%	43%
Anakinra + MTX	Estimate	-23.04	-8.72	-5.83	4.46	10.34	4.86	0	6.30
	95% CrI	(-42.8, -2.06)	(-17.84, 0.37)	(-23.52, 15.01)	(-10.72, 21.41)	(0.25, 20.94)	(-13.21, 22.16)		(-6.65, 20.01)
	P(better)	98%	97%	77%	23%	2%	29%		12%
Tocilizumab + MTX	Estimate	-29.43	-15.06	-12.00	-1.71	4.00	-1.39	-6.30	0
	95% CrI	(-44.66, -13.49)	(-25.14, -5.66)	(-24.98, 2.94)	(-10.29, 7.84)	(-6.97, 14.82)	(-20.05, 16.06)	(-20.01, 6.65)	
	P(better)	>99%	99%	96%	70%	19%	57%	88%	

P(better) = Probability that treatment (in row) is showing greater efficacy than comparator (in column); CrI = credible interval; aTNF = Anti-tumor necrosis factor.

**Table 4:** Treatment effects for all contrast in terms of HAQ-DI along with 95% credible interval and probability that treatment is better than the comparator

**Intervention**		**Comparator**							
		**Placebo**	**MTX**	**aTNF**	**Tocilizumab**	**aTNF + MTX**	**Abatacept + MTX**	**Anakinra + MTX**	**Tocilizumab + MTX**
Placebo	Estimate	0	0.28	0.37	0.53	0.58	0.49	0.39	0.55
	95% CrI		(-0.05, 0.62)	(0.22, 0.53)	(0.27, 0.79)	(0.24, 0.93)	(0.13, 0.87)	(0.02, 0.77)	(0.25, 0.86)
	P(better)		5%	<1%	<1%	<1%	1%	2%	<1%
MTX	Estimate	-0.28	0	0.09	0.25	0.30	0.21	0.11	0.27
	95% CrI	(-0.62, 0.05)		(-0.22, 0.39)	(0.03, 0.47)	(0.22, 0.37)	(0.05, 0.37)	(-0.05, 0.26)	(0.12, 0.42)
	P(better)	95%		27%	2%	<1%	1%	6%	<1%
aTNF	Estimate	-0.37	-0.09	0	0.16	0.21	0.12	0.02	0.18
	95% CrI	(-0.53, -0.22)	(-0.39, 0.22)		(-0.05, 0.37)	(-0.1, 0.52)	(-0.21, 0.47)	(-0.32, 0.36)	(-0.08, 0.44)
	P(better)	>99%	73%		6%	8%	23%	46%	8%
Tocilizumab	Estimate	-0.53	-0.25	-0.16	0	0.05	-0.04	-0.14	0.02
	95% CrI	(-0.79, -0.27)	(-0.47, -0.03)	(-0.37, 0.05)		(-0.18, 0.28)	(-0.3, 0.24)	(-0.41, 0.13)	(-0.14, 0.18)
	P(better)	>99%	98%	94%		32%	63%	88%	39%
aTNF + MTX	Estimate	-0.58	-0.30	-0.21	-0.05	0	-0.09	-0.19	-0.03
	95% CrI	(-0.93, -0.24)	(-0.37, -0.22)	(-0.52, 0.10)	(-0.28, 0.18)		(-0.26, 0.09)	(-0.36, -0.02)	(-0.19, 0.14)
	P(better)	>99%	>99%	92%	68%		85%	98%	65%
Abatacept + MTX	Estimate	-0.49	-0.21	-0.12	0.04	0.09	0	-0.10	0.06
	95% CrI	(-0.87, -0.13)	(-0.37, -0.05)	(-0.47, 0.21)	(-0.24, 0.30)	(-0.09, 0.26)		(-0.33, 0.12)	(-0.16, 0.27)
	P(better)	99%	99%	77%	37%	15%		84%	28%
Anakinra + MTX	Estimate	-0.39	-0.11	-0.02	0.14	0.19	0.10	0	0.16
	95% CrI	(-0.77, -0.02)	(-0.26, 0.05)	(-0.36, 0.32)	(-0.13, 0.41)	(0.02, 0.36)	(-0.12, 0.33)		(-0.06, 0.37)
	P(better)	98%	94%	54%	12%	2%	16%		6%
Tocilizumab + MTX	Estimate	-0.55	-0.27	-0.18	-0.02	0.03	-0.06	-0.16	0
	95% CrI	(-0.86, -0.25)	(-0.42, -0.12)	(-0.44, 0.08)	(-0.18, 0.14)	(-0.14, 0.19)	(-0.27, 0.16)	(-0.37, 0.06)	
	P(better)	>99%	>99%	92%	61%	35%	72%	94%	

P(better) = Probability that treatment (in row) is showing greater efficacy than comparator (in column); CrI = credible interval; aTNF = Anti-tumor necrosis factor.

**Table 5:** Treatment effects for all contrast in terms of SF36-PCS along with 95% credible interval and probability that treatment is better than the comparator

**Intervention**		**Comparator**			
		**MTX**	**Abatacept + MTX**	**aTNF + MTX**	**Tocilizumab + MTX**
MTX	Estimate	0	-4.18	-5.24	-4.58
	95% CrI		(-6.07, -2.27)	(-6.33, -4.16)	(-5.9, -3.27)
	P(better)		<1%	<1%	<1%
Abatacept + MTX	Estimate	4.18	0	-1.08	-0.41
	95% CrI	(2.27, 6.07)		(-3.25, 1.11)	(-2.72, 1.87)
	P(better)	>99%		17%	36%
aTNF + MTX	Estimate	5.24	1.08	0	0.66
	95% CrI	(4.16, 6.33)	(-1.11, 3.25)		(-1.04, 2.36)
	P(better)	>99%	83%		78%
Tocilizumab + MTX	Estimate	4.58	0.41	-0.66	0
	95% CrI	(3.27, 5.9)	(-1.87, 2.72)	(-2.36, 1.04)	
	P(better)	>99%	64%	22%	

P(better) = Probability that treatment (in row) is showing greater efficacy than comparator (in column); CrI = credible interval; aTNF = Anti-tumor necrosis factor.

Both aTNF (-20.2, -17.4, -0.37) and tocilizumab (-31.3, -27.7, -0.53) as monotherapy demonstrated greater reductions in pain, self-reported disease activity (PGA), and HAQ-DI scores than placebo. These improvements over placebo were larger than the MCID for each endpoint.

**Figure 3:**
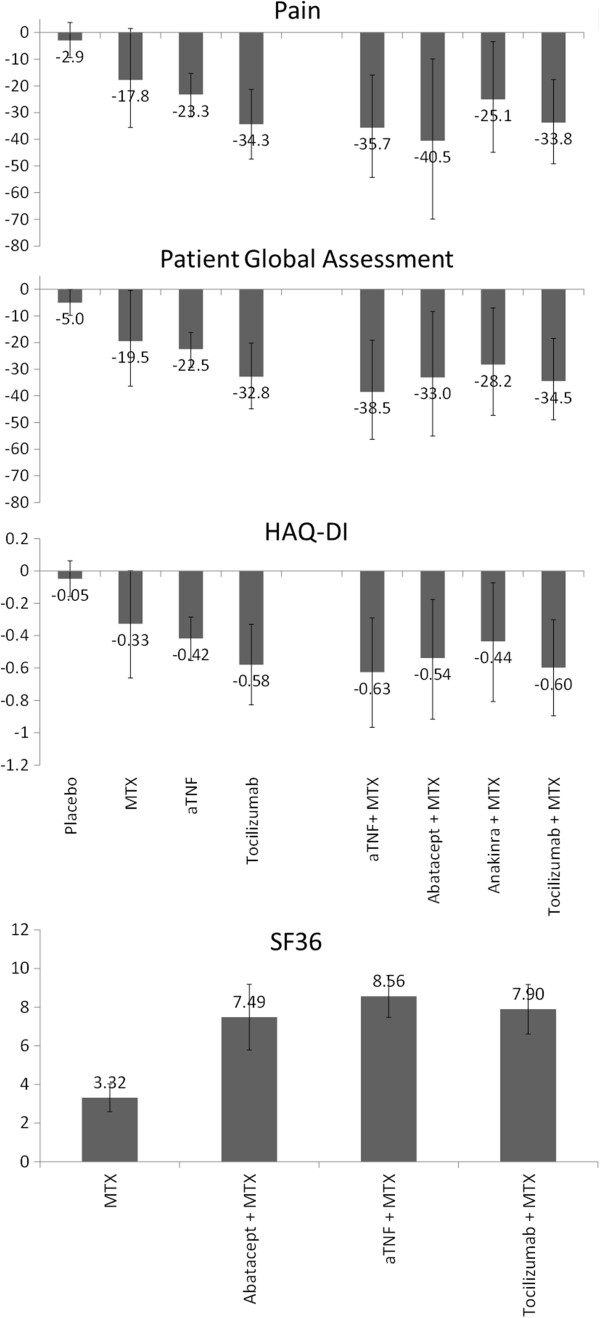
Modeled change in pain, PGA, HAQ-DI and SF36 for different classes of biologic treatments with and without MTX.

Tocilizumab monotherapy showed greater improvements in pain (-11.1; 95% CrI -21.3, -0.1) than aTNF as monotherapy, and can be expected to be more efficacious in terms of PGA as well (-10.3, 95% CrI -20.4, 0.8; probability better = 97%). Tocilizumab was at least as efficacious as aTNF agents in HAQ-DI improvements (-0.16; 95% CrI -0.37, 0.05; probability better = 94%).In Figure 3 the expected reduction in pain, PGA and HAQ-DI for each treatment as monotherapy is presented. Given the available studies, no comparison of SF36 for the biologics as monotherapy was possible.

### Treatment in combination with methotrexate

aTNF (-17.9, -19.1), abatacept (-23.0, -13.6) and tocilizumab (-16.0, -15.1) in combination with MTX showed comparable reductions in pain and PGA relative to MTX in this DMARD-IR population (Tables 2 and 3). These improvements over MTX are expected to be greater than the MCID. The reduction in pain and PGA with anakinra (-7.3, -8.7) was smaller.

Regarding HAQ-DI, the greatest improvements over MTX can be expected with aTNF (-0.30) and tocilizumab (-0.27), both clinically meaningful, followed by abatacept (-0.21) and anakinra (-0.11) (Table 4). Improvements in physical health according to the SF36-PCS with abatacept, aTNF and tocilizumab were comparable (Table 5).

### Comparison of monotherapy and treatment in combination with methotrexate

There is a 93% and 96% probability that aTNF in combination with MTX results in a greater reduction in pain (-12.4) and PGA (-16.1) than aTNF as monotherapy. These differences are expected to be greater than the MCID. For HAQ-DI there is a 92% chance that aTNF with MTX is more efficacious than aTNF as monotherapy (-0.21). For tocilizumab however, the improvement in pain, PGA, and HAQ-DI with and without MTX was comparable at 24 weeks.

**Figure 4:**
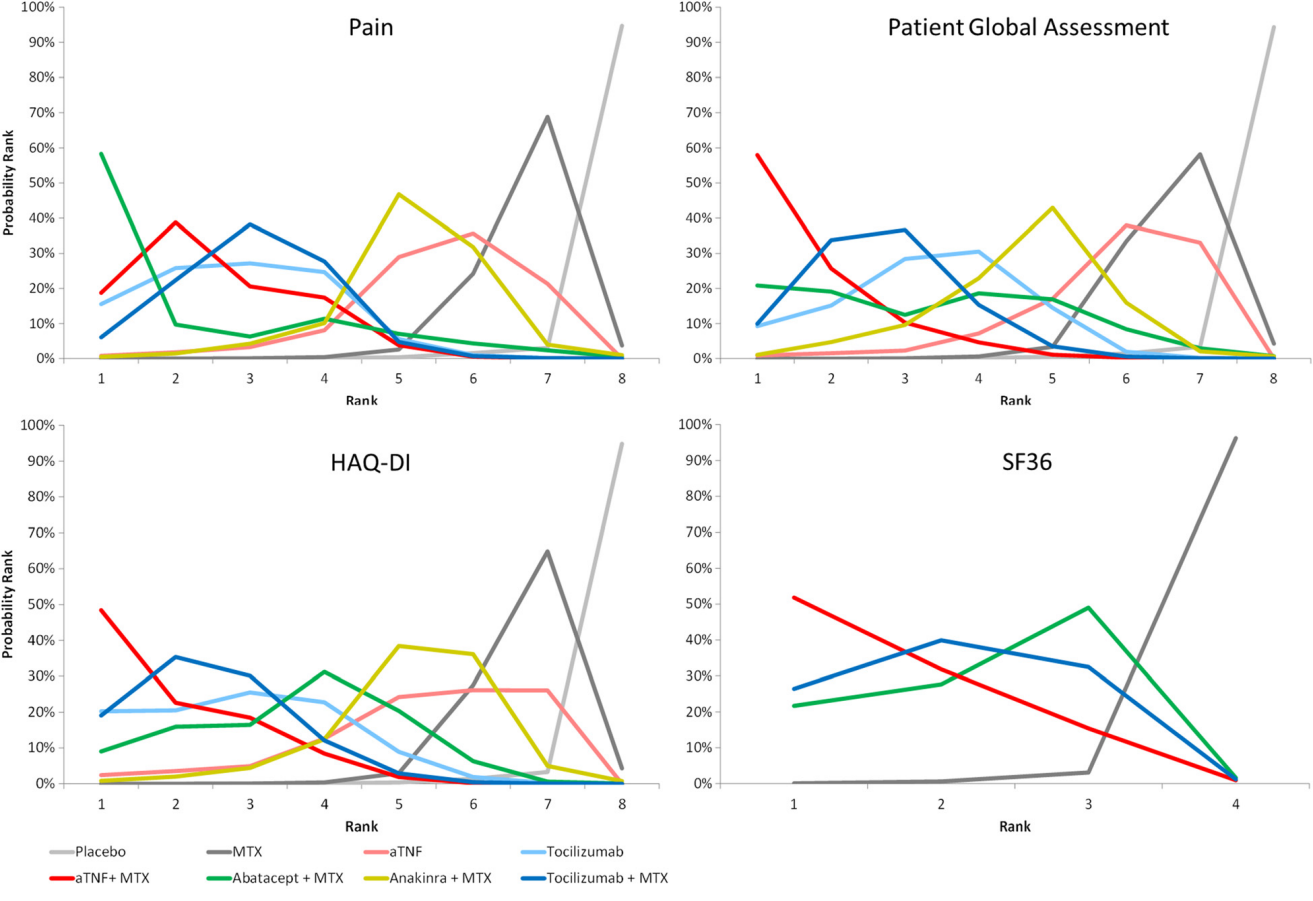
Probability of rank order regarding pain, PGA, HAQ-DI, and SF36 for different classes of biologic treatments with and without MTX.

Figure 4 presents the probability that each intervention is ranked as 1^st^, 2^nd^, 3^rd^ etc. out of all interventions compared for each outcome based on estimated treatment effects and associated uncertainty. These rankograms summarize the available evidence and translate this into measures of decision uncertainty. For example, given the findings in Table 3 there is a 60% probability that aTNFs in combination with MTX result in the greatest PGA improvements, whereas there is <1% probability with aTNF as monotherapy being the best. With aTNF there is ~40% probability that these treatments as monotherapy rank 6 out of all 8 interventions. The ‘shape’ (or distribution) of these rankograms give an idea how well the different interventions are doing. The more the distribution is shifted to the left, the more efficacious the intervention is relative to its competitors. For pain, PGA, and HAQ-DI it can be observed that the rankograms for tocilizumab as monotherapy and in combination with MTX are comparable, whereas the rankograms for aTNF as monotherapy and aTNF in combination with MTX are at opposite ends of the spectrum: tocilizumab as monotherapy and in combination with MTX have a comparable efficacy, whereas aTNF as monotherapy is less efficacious than aTNF with MTX, which is consistent for the three PROs.

## Discussion

RA is a disease that results in a considerable burden for patients due to pain and functional disability [1]. Hence, in addition to effectively treating joint inflammation and reducing the rate of joint deterioration, the aim of treatment is to improve quality of life as well. Since the patient’s perspective on disease outcomes can be different from the physician’s perspective, and the impact of disease on everyday life can only be assessed by the patients themselves, the evaluation of efficacy of interventions for RA should also include PROs. In fact, it has been demonstrated that PROs provide a better discrimination of the impact of treatment effects on symptoms than physician-reported outcomes [57-59].

The objective of this study was to compare the efficacy of different classes of biologic treatments with or without MTX in terms of pain, self-reported disease activity, functional ability, physical and mental health (SF-36) and fatigue among DMARD-IR RA patients. Biologic agents in combination with MTX and as monotherapy were evaluated simultaneously as part of one network of RCTs by means of a network meta-analysis and could therefore be indirectly compared. Both aTNF and tocilizumab as monotherapy demonstrated greater reductions in pain, self-reported disease activity (PGA), and functional ability (HAQ-DI) than placebo. However, improvements with tocilizumab monotherapy were greater than aTNF monotherapy in terms of pain and self-reported disease activity. Tocilizumab was at least as efficacious as aTNF regarding functional ability (HAQ-DI). In combination with MTX, aTNF, abatacept and tocilizumab showed comparable improvements in pain, self-reported disease activity, and physical health as measured with the SF36-PCS component, whereas aTNF and tocilizumab showed the greatest improvements in HAQ-DI. An interesting finding was that aTNFs as monotherapy seem less effective than aTNFs in combination with MTX. With tocilizumab as monotherapy, PROs similar to that of tocilizumab in combination with MTX were observed. The difference between aTNF as monotherapy and aTNF in combination with MTX can be considered clinically meaningful according to the defined MCID for pain (10 mm), PGA (10 mm) and HAQ-DI (0.22).

In addition to pain, self-reported disease activity, functional ability, and physical health, we aimed to perform an analysis for fatigue as well. Fatigue is common in RA [60,61]. Given the differences in fatigue scales used across studies we did not perform a network meta-analysis for this endpoint. However, since fatigue is strongly associated with pain, and secondary associated with disease activity [62], it can be expected to find a similar pattern of efficacy across biologics for fatigue as obtained for pain and PGA.

A limitation of the current analysis is that the study did not explicitly address differences in risk due to adverse events among treatments. However, an analysis of relative short term RCT data would not provide a valid picture of the adverse event risk associated with long-term use of biologics. The evidence of efficacy for all interventions was obtained from RCTs identified by means of a systematic literature review, which is a strength from an internal validity point of view. It is important to realize that the value of randomization holds within trials but not across trials. As such, there is the possibility that differences in study and patients characteristics across studies are modifiers of the treatment effects. This is a source of heterogeneity across studies comparing the same interventions, and a source of bias in the indirect comparison of treatments [29]. There was some variation in duration of disease, lower swollen and tender joint count, and CRP across studies, but we did not observe systematic differences in the distribution of disease duration across different types of direct comparisons. As such, these factors can be a cause of heterogeneity (i.e. variation in true treatment effect across studies within comparison) but are likely not biasing the indirect comparisons. Of course, we can never exclude the possibility of unmeasured differences in patient characteristics across different comparisons.

Although other network meta-analysis of biologic treatments for RA have been published in the past few years [15-22], they focus on clinical outcomes such as the ACR response rates. This is the first network meta-analysis that compares the treatment effects of combination therapy and monotherapy on PROs. This makes it difficult to compare findings, but highlights the value of this review in adding to the evidence base.

In addition to this network meta-analysis of PROs, we recently performed a similar analysis for the ACR 20/50/70 response outcomes. ACR response is a summary measure that captures improvement in tender and swollen joint counts, patient and physician global assessment of disease, pain, C-reactive protein, and disability. The findings of that network meta-analysis were comparable, illustrating that there is not only consistency across the different PROs, but all also with the ACR responses. With the PRO analyses however, the contrasts in efficacy between aTNF as monotherapy and combination therapy seem even stronger. The clinically meaningful differences in pain, PGA and HAQ-DI between monotherapy and combination therapy can have important clinical implications. In patients unable to tolerate MTX, tocilizumab appears to offer a greater likelihood of PRO improvements than aTNF monotherapy and may represent an attractive option in this population.

## Conclusion

Based on a network meta-analysis involving indirect comparison of trial findings, the following can be concluded for DMARD-IR patients: In monotherapy, tocilizumab was associated with greater improvements in pain and self-reported disease activity (PGA) than aTNF, and is at least as efficacious regarding functional ability (HAQ-DI). The efficacy of aTNF, abatacept and tocilizumab in combination with MTX were comparable. Improvements in pain, self-reported disease activity, and functional ability with tocilizumab as monotherapy were similar to that of tocilizumab with MTX, whereas aTNF as monotherapy was likely to be less efficacious than aTNF with MTX.

## Appendix: Search strategy

The following terms were used to search Medline/EMBASE in April 2012:

1. “randomized controlled trial”.pt.

2. (random$ or placebo$ or single blind$ or double blind$ or triple blind$).ti,ab.

3. (retraction of publication or retracted publication).pt.

4. 1 or 2 or 3

5. (animals not humans).sh.

6. ((comment or editorial or meta-analysis or practice-guideline or review or letter or journal correspondence) not “randomized controlled trial”).pt.

7. (random sampl$ or random digit$ or random effect$ or random survey or random regression).ti,ab. not “randomized controlled trial”.pt.

8. 5 or 6 or 7

9. 4 not 8

10. (random$ or placebo$ or single blind$ or double blind$ or triple blind$).ti,ab.

11. RETRACTED ARTICLE/

12. 10 or 11

13. (animal$ not human$).sh,hw.

14. (book or conference paper or editorial or letter or review).pt. not exp randomized controlled trial/

15. (random sampl$ or random digit$ or random effect$ or random survey or random regression).ti,ab. not exp randomized controlled trial/

16. 13 or 14 or 15

17. 12 not 16

18. 9 or 17

19. Arthritis, Rheumatoid/

20. rheumatoid arthritis.ti,ab.

21. 19 or 20

22. (adalimumab or Humira).ti,ab.

23. (etanercept or Enbrel).ti,ab.

24. (infliximab or Remicade).ti,ab.

25. (golimumab or Simponi or CNTO 148).ti,ab.

26. (certolizumab or Cimzia or CDP870).ti,ab.

27. (tocilizumab or Actemra or RoActemra).ti,ab.

28. (rituximab or Rituxan or Mabthera).ti,ab.

29. (abatacept or Orencia or CTLA-4Ig or CTLA-4Ig).ti,ab.

30. (anakinra or Kineret).ti,ab.

31. (tumo?r necrosis factor or TNF).ti,ab.

32. (biologic or biological).ti,ab.

33. 22 or 23 or 24 or 25 or 26 or 27 or 28 or 29 or 30 or 31 or 32

34. 18 and 21 and 33.

The following terms were used to identify trials from the Cochrane Controlled Trials Registry in April 2012:

#1. MeSH descriptor Arthritis, Rheumatoid, this term only

#2. rheumatoid arthritis

#3. (#1 OR #2)

#4. adalimumab or Humira

#5. etanercept or Enbrel

#6. infliximab or Remicade

#7. golimumab or Simponi or CNTO 148

#8. certolizumab or Cimzia or CDP870

#9. tocilizumab or Actemra or RoActemra

#10. rituximab or Rituxan or Mabthera

#11. abatacept or Orencia or CTLA-4Ig

#12. anakinra or Kineret

#13. tofacitinib OR tasaocitinib OR CP-690550

#14. tumo*r necrosis factor OR TNF

#15. biologic or biological

#16. (#4 OR #5 OR #6 OR #7 OR #8 OR #9 OR #10 OR #11 OR #12 OR #13 OR #14 OR #15).

## Abbreviations

aTNF: Tumor necrosis factor blockers; DMARD: Traditional disease-modifying anti-rheumatic drugs; DMARD-IR: Inadequate response to conventional DMARDs; HRQoL: Health related Quality of Life; IL-1: Interleukin-1; IR: Inadequate response; MTX: Methotrexate; PRO: Patient Reported Outcomes; RA: Rheumatoid arthritis; RCT: Randomized controlled trial; HAQ-DI: Health Assessment Questionnaire Disability Index; VAS: Visual analog scale; MCID: Minimum clinically important differences; PCS: Physical health component summary; PGA: Patient’s global assessment of disease activity; MCS: Mental health component summary.

## Competing interests

JP Jansen: Consultant for Genentech; F Buckley: Consultant for Genentech; F Dejonckheere: Employee of Roche; S Ogale: Employee and shareholder of Genentech.

## Authors’ contributions

JJ and FB were responsible for design, systematic review, analysis, interpretation, and writing. FD and SO were responsible for design and writing. All authors read and approved the final manuscript.

## Supplementary Material

Additional file 1: Table S1 Pain, PGA, HAQ-DI and SF36 at 24 weeks as reported in the individual studies used for the network meta-analysis. Click here for file
