# Supplementary material for: Comparative efficacy of biologics as monotherapy and in combination with methotrexate on patient reported outcomes (PROs) in rheumatoid arthritis patients with an inadequate response to conventional DMARDs – a systematic review and network meta-analysis
Source: Health Qual Life Outcomes. 2014 Jul 3;12:102. doi: 10.1186/1477-7525-12-102 (PMC4101713; doi:10.1186/1477-7525-12-102)
Supplement: Additional file 1: Table S1 — Pain, PGA, HAQ-DI and SF36 at 24 weeks as reported in the individual studies used for the network meta-analysis. [file 1477-7525-12-102-S1.doc]

**Additional file 1: Table S1.** Pain, PGA, HAQ-DI and SF36 at 24 weeks as reported in the individual studies used for the network meta-analysis

| **Author, year** | **Intervention** | **Time point (weeks)** | **Pain (VAS)** | | **PGA (VAS)** | | **HAQ-DI** | | **SF36 (PCS)** | |
| --- | --- | --- | --- | --- | --- | --- | --- | --- | --- | --- |
|  |  |  | change from baseline | standard error | change from baseline | standard error | change from baseline | standard error | change from baseline | standard error |
| Kremer 2003 | Placebo + MTX | 26 | -5.48 | 8.42 | -11.05 | 4.86 | -0.14 | 0.10 | 2.89 | 1.91 |
|  | ABT 10mg/kg Q4W+ MTX | 26 | -28.81 | 8.42 | -24.52 | 4.86 | -0.42 | 0.10 | 8.19 | 1.91 |
| Russell 2007 | Placebo + MTX | 24 |  |  |  |  | -0.45 | 0.05 | 4.80 | 0.73 |
|  | ABT 10mg/kg Q4W + MTX | 24 |  |  |  |  | -0.64 | 0.05 | 8.80 | 0.73 |
| Cohen 2004 | Placebo + MTX | 24 | -11.70 | 1.80 | -8.90 | 1.70 | -0.18 | 0.03 |  |  |
|  | ANA 100mg QD + MTX | 24 | -19.00 | 1.70 | -17.70 | 1.60 | -0.29 | 0.03 |  |  |
| Lipsky 2000 | Placebo + MTX | 26 |  |  |  |  | -0.21 | 0.06 |  |  |
| (ATTRACT) | IFX 3mg/kg Q8W + MTX | 26 |  |  |  |  | -0.33 | 0.06 |  |  |
| Keystone 2004 | Placebo + MTX | 24 | -12.60 | 1.85 | -11.40 | 1.99 | -0.24 | 0.04 |  |  |
|  | ADA 40mg QOW + MTX | 24 | -28.20 | 1.79 | -27.20 | 1.87 | -0.56 | 0.04 |  |  |
| Weinblatt 2003 (ARMADA) | Placebo + MTX | 24 | -8.60 | 2.86 | -8.60 | 3.19 | -0.27 | 0.07 |  |  |
|  | ADA 40mg QOW + MTX | 24 | -25.10 | 4.04 | -29.70 | 3.88 | -0.62 | 0.08 |  |  |
| Van de Putte 2004 | Placebo | 26 | -11.00 | 2.55 | -10.60 | 2.65 | -0.07 | 0.05 |  |  |
|  | ADA 40mg QOW | 26 | -27.60 | 2.93 | -27.90 | 2.87 | -0.38 | 0.06 |  |  |
| Strand 2009 (RAPID 1) | Placebo + MTX | 24 | -8.20 | 4.56 | -8.18 | 4.59 | -0.17 | 0.09 | 1.80 | 0.60 |
|  | CTZ 200mg QOW + MTX | 24 | -29.50 | 4.56 | -29.60 | 4.59 | -0.58 | 0.09 | 7.70 | 0.40 |
| Smolen 2009 (RAPID 2) | Placebo + MTX | 24 | -4.70 | 1.90 | -4.20 | 2.00 | -0.14 | 0.04 | 0.90 | 0.68 |
|  | CTZ 200mg QOW + MTX | 24 | -23.70 | 1.40 | -24.50 | 1.40 | -0.50 | 0.03 | 5.20 | 0.53 |
| Fleischmann 2009 | Placebo | 24 | 1.70 | 4.75 | 0.00 | 3.01 | 0.13 | 0.10 |  |  |
| (FAST4WARD) | CTZ 400mg Q4W | 24 | -20.60 | 4.75 | -14.14 | 3.01 | -0.36 | 0.10 |  |  |
| Weinblatt 1999 | Placebo + MTX | 24 | -12.00 | 4.82 | -20.00 | 5.08 | -0.40 | 0.06 |  |  |
|  | ETN 25mg BW + MTX | 24 | -32.00 | 3.44 | -40.00 | 3.62 | -0.70 | 0.06 |  |  |
| Mathias 2000 | Placebo | 26 | 14.30 | 10.58 | 2.07 | 7.28 | -0.14 | 0.16 | * |  |
|  | ETN 25mg BW | 26 | -35.51 | 10.58 | -32.20 | 7.28 | -0.57 | 0.16 |  |  |
| Keystone 2009 (GO-FORWARD) | Placebo + MTX | 24 |  |  |  |  | -0.13 | 0.04 |  |  |
|  | GLB 50mg Q4W + MTX | 24 |  |  |  |  | -0.38 | 0.05 |  |  |
| Genovese 2008 | Placebo + MTX | 24 |  |  |  |  | -0.20 | 0.05 | 4.10 | 0.87 |
| (TOWARD) | TCZ 8mg/kg Q4W + MTX | 24 |  |  |  |  | -0.50 | 0.05 | 8.90 | 0.87 |
| Smolen 2008 (OPTION) | Placebo + MTX | 24 | -14.00 | 2.13 | -17.80 | 2.16 | -0.34 | 0.08 | 5.00 | 0.57 |
|  | TCZ 8mg/kg Q4W + MTX | 24 | -29.80 | 2.13 | -32.70 | 2.16 | -0.55 | 0.07 | 9.50 | 0.56 |
| ACT-RAY | TCZ 8mg/kg Q4W + MTX | 24 | -29.34 | 1.67 | -34.31 | 1.61 | -0.56 | 0.04 |  |  |
|  | TCZ 8mg/kg Q4W | 24 | -29.75 | 1.59 | -32.42 | 1.55 | -0.54 | 0.03 |  |  |
| ADACTA | ADA 40mg | 24 | -28.70 | 2.53 | -31.80 | 2.60 | -0.52 | 0.06 | * |  |
|  | TCZ 8mg/kg | 24 | -40.10 | 2.53 | -42.30 | 2.60 | -0.68 | 0.06 |  |  |

VAS=visual analogue scale; ABT=abatacept; ANA=anakinra; IFX=infliximab; ADA=adalimumab; CTZ=certolizumab pegol; ETN=etanercept; GLB=golimumab; TCZ=tocilizumab; MTX=methotrexate
*Study has SF36 (PCS) results available, but could not be used in network meta-analysis because no link with other trial.
